# Immunization Gender Inequity in Pakistan: An Analysis of 6.2 Million Children Born from 2019 to 2022 and Enrolled in the Sindh Electronic Immunization Registry

**DOI:** 10.3390/vaccines11030685

**Published:** 2023-03-17

**Authors:** Danya Arif Siddiqi, Sundus Iftikhar, Muhammad Siddique, Mariam Mehmood, Vijay Kumar Dharma, Mubarak Taighoon Shah, Hamidreza Setayesh, Subhash Chandir

**Affiliations:** 1 IRD Global, Singapore 049145, Singapore; 2 IRD Pakistan, Karachi 75190, Pakistan; 3 Gavi, The Vaccine Alliance, 1218 Geneva, Switzerland

**Keywords:** gender inequity, routine immunization, male-to-female ratio, female vaccination, timeliness of immunization

## Abstract

Gender-based inequities in immunization impede the universal coverage of childhood vaccines. Leveraging data from the Government of Sindh’s Electronic Immunization Registry (SEIR), we estimated inequalities in immunization for males and females from the 2019–2022 birth cohorts in Pakistan. We computed male-to-female (M:F) and gender inequality ratios (GIR) Tfor enrollment, vaccine coverage, and timeliness. We also explored the inequities by maternal literacy, geographic location, mode of vaccination delivery, and gender of vaccinators. Between 1 January 2019, and 31 December 2022, 6,235,305 children were enrolled in the SEIR, 52.2% males and 47.8% females. We observed a median M:F ratio of 1.03 at enrollment and at Penta-1, Penta-3, and Measles-1 vaccinations, indicating more males were enrolled in the immunization system than females. Once enrolled, a median GIR of 1.00 indicated similar coverage for females and males over time; however, females experienced a delay in their vaccination timeliness. Low maternal education; residing in remote-rural, rural, and slum regions; and receiving vaccines at fixed sites, as compared to outreach, were associated with fewer females being vaccinated, as compared to males. Our findings suggeste the need to tailor and implement gender-sensitive policies and strategies for improving equity in immunization, especially in vulnerable geographies with persistently high inequalities.

## 1. Introduction

Vaccination is considered one of the most successful and cost-effective interventions in public health, with a potential return on investment of up to USD 16 per dollar spent [1]. However, many countries, particularly low- and middle-income countries (LMICs), struggle to equitably vaccinate all children, leading to persistent immunization inequities across multiple socio-demographic dimensions, with gender-based inequities being a prominent factor [2]. Although there are apparently no significant differences in coverage rates between males and females at the global level, several country-specific studies have provided contrary evidence [3]. Studies have shown there were significant biases in immunization coverage rates that disadvantaged females in South and Southeast Asia, with Pakistan reporting a 7.8 percentage-point difference between males and females in terms of complete immunization; Cambodia reporting a difference of 4.9 percentage points; Nepal, a difference of 4.3 percentage points; and India, with the largest gap of 13.4 percentage points [4]. In addition to varying inequities at the country level, substantial differences also exist within countries, highlighting an interplay of complex socio-cultural, economic, and geographic factors that leave females at a disadvantage when accessing immunization services.

Pakistan is among the countries where gender inequity in immunizations is a growing concern. As per the Global Gender Gap Index Report 2022 [5], the country ranked 143 out of a total of 146 countries for health and survival, highlighting the adverse position of females relative to males, with inequities manifesting in areas such as healthcare and immunizations. The Pakistan Demographic and Health Survey (2017–18) [6] showed there was a significant difference in coverage rates between females and males, with females being less likely to receive all basic vaccinations, as compared to males (63% vs. 68%), eventually contributing to higher morbidity and mortality among females over the long term. Although concerted efforts in recent decades have resulted in improved immunization coverage rates in the country overall [7], the trend of differential coverage rates among females and males remains, underscoring the gaps in equitable coverage. This is partly due to the lack of gender-sensitive immunization strategies, which are difficult to design in the face of the unavailability of gender-disaggregated data at the micro-level. This has led to a lack of evidence regarding the true estimates and the extent of immunization inequities in the regions where females are most likely to fall behind males. Additionally, there is insufficient information regarding the risk factors associated with unequal coverage rates, and understanding of the demand- and supply-side barriers that consistently prevent females from accessing immunizations. 

Major global immunization initiatives, including the Immunization Agenda 2030 and the Gavi 5.0 strategy, were designed around the themes of “Leave No One Behind” and “endeavor to reach the furthest behind first” [8], highlighting the need for identifying, understanding, and addressing the gender-related barriers to immunizations. It is critically important for governments and other stakeholders to estimate the true extent of female-based gender inequities in immunization outcomes at a micro-geographic level and delineate the contributing factors. It is also vital to identify the supply-side barriers that can adversely impact immunization uptake by females. This crucial information is important for immunization systems to implement targeted approaches for reaching missed female children, ensuring their immunization completion as per the WHO-recommended immunization schedule, and promoting gender-based equity in immunizations.

We leveraged the individual child-level data from the Government of Sindh’s Electronic Immunization Registry (SEIR) to uncover a detailed picture of the gender inequities in childhood immunizations. We estimated the male-to-female ratios for coverage and timeliness at the micro-geographic level by districts and union councils (UCs; smallest geographic administrative unit) in Sindh Province, Pakistan. Additionally, we also examined the gender inequality ratios for the above as an additional measure. We examined how maternal literacy levels, geographic area (urban, rural, remote-rural, and slum areas), and supply-side factors (gender of vaccinators and modality of immunization service delivery) affect gender inequities in immunization.

## 2. Methods

### 2.1. Population

As per the population estimates for 2022, Sindh Province has an annual birth cohort of 1.9 million [9], and a total population of 53.8 million people, with a population density of 381.1 people/sq. km [10]. The province comprises 6 divisions, which are further divided into 30 districts with 1130 UCs [11]. The median population of the UCs is 46,401 (range: 8371–574,2572). The urban and rural median populations of the UCs are 59,293 (range: 8371–574,257) and 37,936 (range: 13,000–95,886), respectively. The poverty index of the province is 0.28 (district range: 0.02–0.50) [12]. The literacy rate for the province is 58% (male = 68%; female = 47%; urban = 73%; rural = 39%) [13]. The annual target population (0–23-month-old children) for the Expanded Programme on Immunization (EPI) was 1.9 million in 2022. Immunizations in Sindh are administered predominantly through public services supplemented by private clinics [14]. Traditionally, approximately 60% of all provincial immunizations were provided through fixed immunization centers, whereas the rest were delivered through routine outreach sessions [15]. However, after the COVID-19 pandemic, this proportion has reversed, with almost 60% of the immunizations now being provided through outreach [16]. Routine outreach comprises immunization sessions held at a site other than the immunization center, from which vaccinators can go out and return the same day, whereas enhanced outreach is defined as a series of immunization outreach sessions covering a geographic area outside the radius of routine activities [17]. 

### 2.2. Data Source

We used geospatial-enabled immunization records from the SEIR (also known as Zindagi Mehfooz *(Safe Life)* Electronic Immunization Registry; ZM-EIR). SEIR is an Android-based application that allows vaccinators to enroll and track children’s immunization records. The SEIR captures routinely collected data, including the child’s demographic details (child’s name, father’s name, caregiver national identity card number (optional), and contact information) and immunization details (vaccination status, dates, and modality). Additionally, the SEIR also captures the health facility and vaccinator details and the geolocation of each vaccination. Each child’s record is tracked through a unique identifier assigned to the child at the time of enrollment in the SEIR. Performance management of the data of vaccinators, including attendance and compliance of usage of the system, is also captured.

The SEIR was scaled up in October 2017 across 28 districts of Sindh and was later rolled out to the remaining 2 districts, Khairpur and Dadu (where primary health care is delivered through a public–private partnership) on 24 February 2020 and 29 June 2020, respectively. Currently, the SEIR is being used across all 30 districts of Sindh, by 3565 vaccinators (including 15.0% female vaccinators) working at 1785 public and 373 private immunization clinics. As of 31 December 2022, the SEIR enrolled >7.7 million children and >2.6 million females and recorded >90 million immunization events. The SEIR enrolled 108.34%, 96.49%, 97.26%, and 95.34% of the EPI estimated annual birth cohorts of 2019, 2020, 2021, and 2022, respectively (1,340,207; 1,638,386; 1,642,773; and 1,682,569, respectively), in the districts where it was operational.

### 2.3. Study Design and Procedure

We analyzed the child-level longitudinal immunization records in the SEIR from 1 January 2019 to 31 December 2022, for all 30 districts of Sindh. Data from District Khairpur and District Dadu were not shown for children who had received their vaccinations in 2019, as the SEIR was launched in these districts in 2020. We extracted data related to the demographic profile (gender, age, and maternal literacy level), immunization history (vaccines, date of administration, and geo-coordinates of vaccine administration site); modality of immunization service delivery (fixed, routine outreach, or enhanced outreach), and geographical location of household (district, UC, urban vs. rural area, rural vs. remote-rural area and slums vs. non-slums) of children from the 2019–2022 birth cohorts enrolled in the SEIR. Out of 1130 UCs in the province, 464 were classified as urban, and 666 as rural. Within the rural UCs, 88 were classified as remote-rural UCs, and within the urban UCs, 89 were classified as slum areas. An slum area was defined as a contiguous settlement where the inhabitants are characterized as having inadequate housing and basic services. Slum UCs were defined as having >75% population living in poverty. The slum area analysis was limited to EPI-identified slums in the eight districts of Karachi and Hyderabad [18]. All slum UCs in Karachi and Hyderabad were in urban areas. Remote-rural UCs were classified according to the Government of Sindh’s School Education and Literacy Department classification of hard-area UCs that were located in remote coastal, desert, or mountainous areas [19]. Remote-rural UCs were mostly concentrated in the eastern and western peripheries of the province; urban UCs were found within the cities of Karachi and Hyderabad; and the rest of the remaining UCs in the province were predominantly rural (Supplementary, Figure S1). In addition to the geo-location data, we also extracted the gender profile of vaccinators who used the SEIR across the province. 

### 2.4. Vaccination Schedule 

Pakistan’s routine immunization schedule included the following vaccines: BCG (Bacille Calmette-Guérin) and oral polio vaccine (OPV) vaccine at birth; 3 doses of pentavalent (DPT, HepB, Hib) vaccine; 3 doses of pneumococcal conjugate vaccine (PCV) and 3 doses of OPV at 6, 10, and/or 14 weeks of age; 2 doses of rotavirus vaccines at 6 and 10 weeks of age; 2 doses of inactivated polio vaccine (IPV) at 14 weeks and 9 months of age; and 2 doses of measles–rubella vaccine and typhoid conjugate vaccine (TCV) at 9 and 15 months of age. TCV, second dose of IPV, and rubella vaccine were added to the EPI schedule on 1 January 2020, 3 May 2021, and 15 November 2021, respectively [20].

### 2.5. Ethics

This analysis was deemed to be exempt by the Institutional Review Board of Interactive Research and Development under 45 CFR 46.101(b). The IRB was registered with the U.S. Department of Health and Human Services Office for Human Research Protections with registration number IRB 404 00005148.

### 2.6. Outcome

The primary outcome was the male-to-female ratios (M:F) at enrollment and by antigens among children from the 2019, 2020, 2021, and 2022 birth cohorts enrolled in the SEIR. The M:F ratio was the number of vaccinated males relative to females. Enrollment was defined as the first encounter of the child with the SEIR. We calculated the M:F ratios at the district and UC levels. We adjusted the M:F ratios using the sex ratios at birth (1.055) in Pakistan [21]. We computed the M:F ratios for the up-to-date vaccination coverage for Pentavalent-1, Pentavalent-3, and Measles-1 at 6, 9, 12, 18, and 24 months. Up-to-date coverage was defined as the proportion of 0–24 months children who received vaccinations by the specified months of age. In order to examine timely coverages, we also calculated the up-to-date coverage of Penta-1 at 10 weeks, Penta-3 at 18 weeks, and Measles-1 at 10 months to account for the timeliness criteria used by EPI-Sindh (an additional 4 weeks’ time duration beyond the age at which each vaccine is due, as per the WHO-specified EPI schedule). Furthermore, we compared the M:F ratios by maternal literacy level, geographic residential location of the child (urban vs. rural, rural vs. remote-rural and slums vs. non-slums), modality of vaccination (fixed center, outreach, and enhanced outreach), and the sex ratio of vaccinators in the province. As a secondary outcome, we also calculated the Gender Inequality Ratio (GIR) for all the above analyses, where the gender inequality ratio was defined as the proportion of vaccinated males among those who were due for vaccination, relative to the proportion of vaccinated females who were due for vaccination.

### 2.7. Statistical Analysis

We reported the median and interquartile range (IQR) of the UCs for the M:F ratios, along with the ranges at the UC level. UCs with no children vaccinated for any particular vaccine were excluded from the analysis for that particular vaccine only. A male-to-female ratio of 0.00 indicated that there were no males or females vaccinated in the particular UC. This was due to the reduced population sizes when we examined our indicators across the sub-categories (maternal literacy and geographic location of vaccination) within a UC.

For our secondary outcome, we computed the GIR by dividing the proportion of males who were due and received vaccinations by the proportion of females who were due and received vaccinations. A GIR of 1.00 implied no differential in coverage rates between females and males, whereas a GIR of above 1.00 indicated inequalities (with higher coverage rates for males relative to females). We performed statistical analyses with Stata, release 17 (StataCorp, College Station, TX, USA). We used digital maps to review the immunization coverage by the district and UC using QGIS (3.16.7-Hannover).

## 3. Results

Between 1 January 2019, to 31 December 2022, a total of 6,235,305 children were enrolled in the SEIR from the 2019 (23.29%), 2020 (25.35%), 2021 (25.62%), and 2022 (25.73%) birth cohorts. The proportion of males enrolled in the SEIR, as compared to females, was consistently higher across all birth cohorts (2019: 52.11%, 2020: 52.14%; 2021: 52.30%; 2022: 52.25%) (data not shown).

Across districts, we found a distinctive pattern in districts Kashmore, Ghotki, Jacobabad, and Tharparkar, having the highest adjusted median M:F ratios at enrollment: (Kashmore: 1.11 (IQR: 1.04–1.25); Ghotki: 1.11 (IQR: 1.04–1.16); Jacobabad: 1.07 (IQR: 1.00–1.13), and Tharparkar: 1.12 (IQR: 1.02–1.18)) (Table 1). The findings were similar for Penta-1, Penta-3, and Measles-1 vaccinations. A consistent trend, therefore, emerged, showing females falling behind males consistently in these districts from enrollment into the SEIR until their Measles-1 vaccination. At the UC-level, a high median M:F ratio emerged for selected UCs in District Thatta, where thrice the number of males were vaccinated, as compared to females.

When examining the GIR, we observed that once children were enrolled in the SEIR, coverage rates for vaccines were similar for females and males, as shown by the UC-level median GIR for Penta-1 (median: 1.00, IQR: 1.00–1.01), Penta-3 (median: 1.00, IQR: 0.99–1.01), and Measles-1 (median: 1.00, IQR: 0.99–1.01) (Supplementary Table S1).

Tracking the M:F ratios for vaccines over the 4 years showed high inequities in the number of females vaccinated, as compared to males, in 2019 for Penta-3 (1.14, range: 0.24–8.00) and Measles-1 (1.14, range: 0.14–5.00), which declined to 1.10 (Penta-3 range: 0.49–5.00; Measles-1 range: 0.25–2.07) in 2020 and remained at the same level for the following 2 years. The M:F ratios for Penta-1 remained roughly the same between 2019 and 2022, showing no major progress was made in reducing these disparities over the last 4 years (Supplementary Figure S2). The GIR reflected a similar picture of slightly higher inequalities in coverage among the enrolled children in 2019. Thereafter, coverage rates became more balanced between females and males (GIR: 1.00) for all the vaccines in 2020–2022 (Supplementary Figure S3). At the UC level, we found that 11.6% (131/1129) of the UCs showed a M:F > 1.10 for Penta-1 consistently over the four years. This proportion was 10.7% (121/1129) for Penta-3 and 8.9% (101/1129) for Measles-1, reflecting certain geographic pockets had persistently higher numbers of males being vaccinated, as compared to females, year-on-year (Supplementary Table S3). A closer geographic examination revealed that these UCs were spread throughout the province, as opposed to being located in clusters (Supplementary Figure S4).

**Figure 1:**
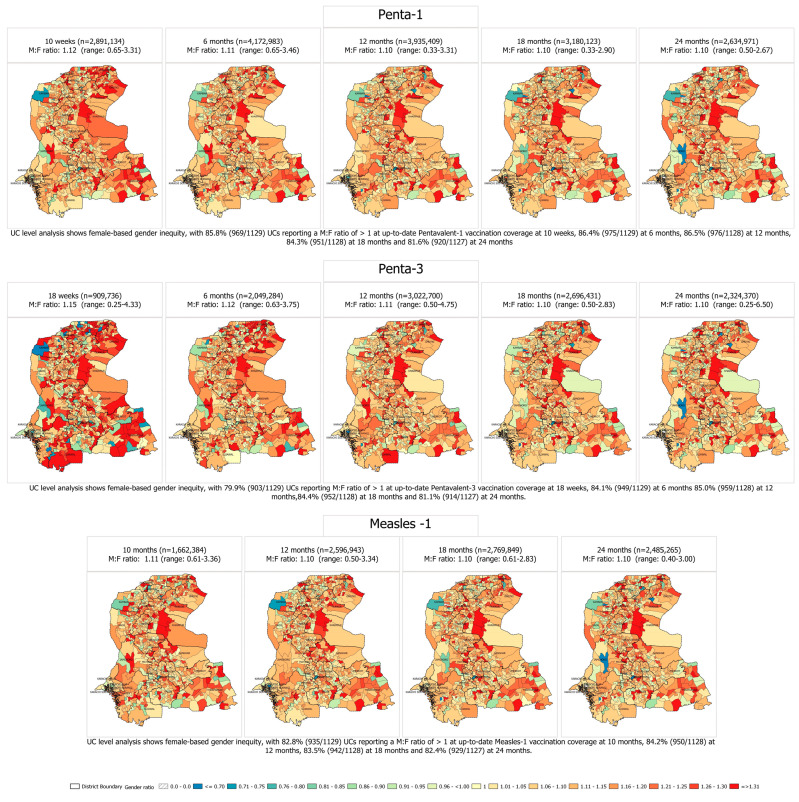
Male-to-female ratios of up-to-date vaccination coverage of Pentavalent-1 at 10 weeks and 6, 12, 18, and 24 months; Pentavalent-3 at 18 weeks and 6, 12, 18, and 24 months; and Measles-1 at 10, 6, 12, 18, and 24 months, in 0–23-month-old children in 2019–2022 birth cohorts enrolled in SEIR (1 January 2019–31 December 2022).

The up-to-date coverages at specific age intervals for Penta-1, Penta-3, and Measles-1 showed more males were vaccinated, as compared to females, at each age (M:F ≥ 1.10) (Figure 1).

**Figure 2:**
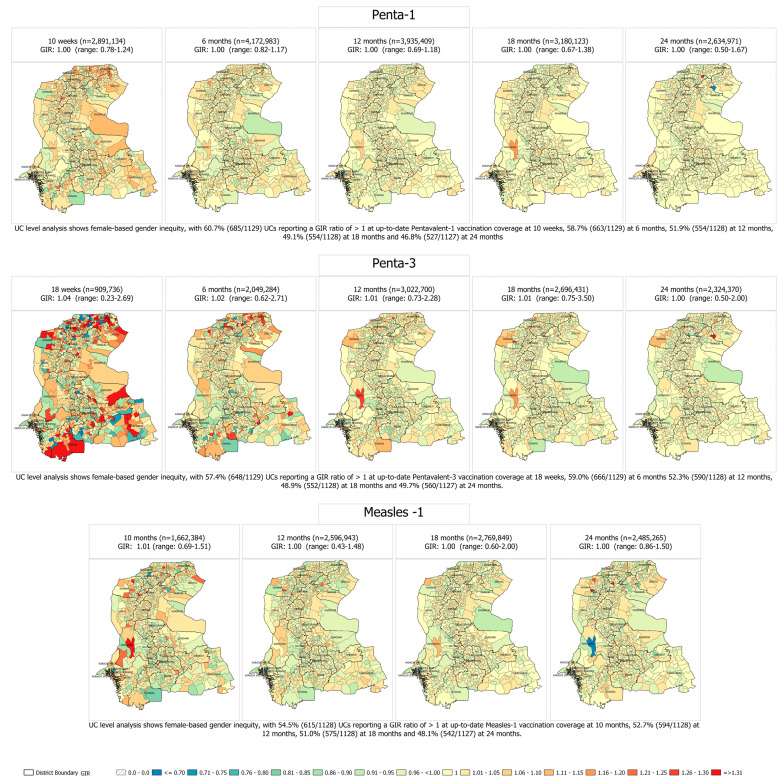
Gender inequality ratio (GIR) at up-to-date vaccination coverage of Pentavalent-1 at 10 weeks and 6, 12, 18, and 24 months; Pentavalent-3 at 18 weeks and 6, 12, 18, and 24 months; and Measles-1 at 10, 6, 12, 18, and 24 months, in 0–23-month-old children in 2019–2022 birth cohorts enrolled in SEIR (1 January 2019–31 December 2022).

Among the enrolled children, 1 out of every 2 UCs in the province had females falling behind males on timely vaccinations of Penta-1, Penta-3, and Measles-1, as denoted by GIRs > 1.00. Notably, 60.7% (685/1129), 57.4% (648/1129), and 54.5% (615/1128) of UCs had GIRs > 1.00 for up-to-date coverage of Penta-1 at 10 weeks, Penta-3 at 18 weeks, and Measles-1 at 10 months. This proportion continued to decline across ages, demonstrating a narrowing of the inequity gap at the UC level as children aged (Figure 2).

By observing the inequities in enrollment and the number of vaccinated males and females across maternal literacy levels, we found higher inequities among children with mothers who had only primary education (1–5 years of education), as compared to mothers with higher education levels and those who were not educated at all (Table 2). This was evident for Penta-1 (median M:F ratio: 1.09 (IQR: 0.92–1.3)), Penta-3 (median: 1.10 (IQR: 0.91–1.33)), and Measles-1 (median: 1.10 (IQR: 0.93–1.33)). With increasing education levels, the inequities were reduced, as shown by the median M:F ratio declining to 1.00. However, when examining the inequities at the UC level, individual UCs had high inequities in enrollment and the number of vaccinated males vs. females (M:F ratio between 7.00–10.00), even when mothers had high literacy levels (≥11 years of education).

Rural UCs had higher median M:F ratios, as compared to urban UCs, for Penta-1 (median M:F ratio 1.11 vs. 1.06), Penta-3 (M:F ratio: 1.11 vs. 1.06), and Measles-1 vaccinations (M:F ratio: 1.10 vs. 1.07). The UC-level ranges, however, demonstrated that there were selected UCs with as many as five times more males being vaccinated than females for Measles-1, even in urban areas. Within the rural UCs, the remote-rural UCs reflected worse equity outcomes, with median M:F ratios as high as 1.14 for Penta-1. The slum UCs had the worst median M:F ratios for Penta-1 (1.07 (IQR: 1.03–1.11)), Penta-3 (1.07 (IQR: 1.03–1.12)), and Measles-1 (1.07 (IQR: 1.03–1.12)), as compared to non-slum UCs (Penta-1: 1.05 (IQR: 1.01–1.09), Penta-3: 1.05 (IQR: 1.02–1.09), and Measles-1 1.06 (IQR: 1.01–1.09).

Based on M:F ratios by mode of vaccination, we found marginally higher inequities in the number of males vaccinated, as compared to females, among vaccinations conducted at fixed immunization centers, as compared to immunizations by routine outreach (Penta-1: 1.09 vs. 1.08; Measles-1: 1.09 vs. 1.08)). Lower median M:F ratios were found for immunizations administered by EOAs, (Penta-1: 1.07; Penta-3: 1.07; and Measles-1: 1.07), showing more equity between the number of females and males vaccinated during the intensive periods of EOAs conducted in the province.

Slight variations in M:F ratios were also observed when investigating inequities across UCs with varying numbers of female and male vaccinators. No differences between the number of males and females vaccinated (across any antigen) were observed when examining the median M:F ratios. However, we observed slightly increased inequities at the UC level in areas where there were no female vaccinators (UC range: 0.80–3.00 for Penta-1 and 0.80–2.80 for Penta-3 and Measles-1). We noted that even in areas where there were more female than male vaccinators (selected UCs in Karachi Division, Supplementary Figure S5), there were UCs that still had fewer females vaccinated than males (UC range: 1.00–1.20).

Conducting the above analysis according to the GIR did not reveal substantial inequalities in coverage rates between males and females (median GIR of UCs ranged between 0.99–1.03). Selected UCs demonstrated high inequalities. Nevertheless, a clear correlational pattern between inequality in coverage and maternal literacy, geographic location, modality of vaccination, and sex ratio of vaccinators, was not always obvious (Supplementary Table S2).

## 4. Discussion

We found that for every 100 females, 103 males were enrolled and vaccinated in the SEIR over the last 4 years. However, the sub-national analysis at the UC level shows the difference increased to 300 males being vaccinated for every 100 females in specific UCs. Merely observing the aggregate levels for evidence of gender differentials masked these nuanced yet more pronounced inequities. Moreover, recent reports by Gavi [22] and WHO [23] asserted that subnational variations in immunization coverage were ‘one of the tractable but unfinished challenges of immunization inequity globally.’ Differences at the micro-geographic level reflected subtle and persistent forms of gender bias and discrimination that continue to affect health outcomes for females over the long term. When comparing the male-to-female ratios and gender inequality ratios, we observed a larger number of males than females made contact with the immunization system (even after adjusting for the male-to-female baseline population). However, once they had been enrolled (in the SEIR), the vaccine coverage rates were similar for both females and males, although females still fell behind males in receiving timely vaccinations.

Our findings have important implications for the zero-dose children that have yet to make contact with the health system. Since more males than females have been enrolled in the immunization system, this reflects substantial inequities, indicating more females than males are left behind and being added to the higher proportion of zero-dose children. There is a need for rethinking and emphasizing the narrative of ‘zero-dose females’, and ensuring the use of gender-disaggregated data and gender-sensitive strategies in order to reach the missing children. We also observed that gender inequities continue to persist over time. The analysis of individual UCs suggested there were certain pockets and regions spread throughout the province where females continuously fell behind males on their vaccinations, year-on-year. Targeted, intensified efforts directed to hotspots showing high inequities could be a potential measure to break the pattern of persistent inequities.

Although parity in coverage rates among females and males enrolled in the SEIR was a positive finding, we observed equality was not uniformly reflected across all age groups. Females were more likely to be delayed in their vaccinations than males. While reflecting well on the overall view of equality, it was imperative to note that as females were delayed on their vaccination, they remained susceptible to vaccine-preventable diseases (VPDs) for longer periods, leading to a higher risk of morbidity and mortality over time. Delayed vaccination for females could have a considerable impact on child survival rates overall, a pertinent implication for Pakistan, where infant and child mortality rates are some of the highest globally. A study from Bangladesh showed children receiving BCG within the first 6 months of life had a lower risk of diseases than those vaccinated later [24]. Similar results were also reported for the delayed administration of the diphtheria–tetanus and pertussis vaccines [25].

Our findings of higher inequities in the number of vaccinated females and males in rural areas, as compared to urban areas, and slums, as compared to non-slums [26,27], have been repeatedly emphasized in existing literature [28,29]. We went a step further to demonstrate that within the rural areas, the category of remote-rural and hard-to-reach areas fared even worse, with M:F ratios as high as 1.14. Several underlying factors have been cited to explain the inequities, the most prominent being the deep-rooted socio-cultural practice of “son preference”, which is inherently common in Pakistan [30] and other South Asian countries [29,31]. Persistent patriarchal practices favor sons over daughters due to factors such as carrying forward the family lineage, providing old-age support, financial support, and practices pertaining to dowries. The phenomenon of son preference has been closely associated with several adverse practices, including gender-selective abortions, female infanticide, and neglect of the health and education of females. In rural and remote-rural regions, not only are these practices more deeply entrenched, but when coupled with multiple other deprivations, including poverty, lack of affordable transportation, and long distances to healthcare services, they lead to discriminatory attitudes by caregivers in favor of males. This was underscored in our findings with higher inequities in the number of females and males vaccinated at fixed immunization centers. Immunizations administered during both routine and enhanced outreach tended to be more equitable for females, reflecting that caregivers were not inherently opposed to vaccination, but when faced with the logistical and financial challenges of taking children to vaccination centers, they were more likely to favor males over females.

Within remote-rural settings, several additional dynamics are at play that adversely impacted equitable immunization, such as the higher marginal cost of reaching remote children, retention and motivation of personnel, geographic remoteness, and limited socio-political power among communities [32]. The factors were further undercut by gender issues where, in the event of male vaccinators, mothers and female caregivers faced even greater societal restrictions when accompanying children for immunization. Our findings showed that not only did this have an adverse impact on vaccination rates overall, but the lack of female vaccinators disproportionately and adversely affected vaccination outcomes for females, as compared to males. The absence of gender-sensitive policies for immunization was highlighted in our study (none of the 87 remote-rural UCs in Sindh Province had a single female vaccinator (Supplementary Table S4) and mentioned elsewhere including no segregated waiting rooms at immunization facilities for female caregivers and a shortage of female vaccinators in urban impoverished areas, which was a “discouraging factor” for the attendance of females and children at health facilities [26]. Our results showed that the districts of Ghotki, Jacobabad, and Kashmore had high prevalence rates of inequities for females at enrollment and for subsequent antigens. These districts are located within the northern belt of the province, which remains deeply rooted in conservative tribal culture with a high prevalence of other discriminatory practices against females, including domestic violence and forced child marriages [33]. Gender equity in immunizations is not an isolated concept but deeply intertwined with females’ empowerment, agency, and autonomy. Increasing females access to education is a proven mechanism to break the perpetual cycle of discrimination. Within the context of immunizations, our findings were in line with others that showed higher maternal education [34,35] led to reduced vaccination inequities for females. However, our study showed that, even with very high levels of maternal education (>11 years), there remained UCs that had extreme inequalities (M:F ratio: 11.0). Upon closer geographic examination, we observed 4 of such UCs were clustered fairly close together, suggesting there could be other prevalent socio-cultural or logistical challenges causing inequities that even higher maternal education levels were unable to overcome. Vaccine hesitancy is one particular challenge that merits further investigation within the context of gender inequities in immunization. One study has articulated the reasons for vaccine hesitancy in Pakistan as a triad of religious traditions, misconceptions, and political factors. [36]. Vaccine hesitancy may contribute to gender inequities in immunization by perpetuating cultural norms and beliefs that prioritize males over females and fuel misinformation and misconceptions about vaccines that disproportionately affect females, limiting access to health services and decision-making power for females.

Addressing gender inequities in immunization requires multilevel, complementary approaches. Feasible policy measures include the inclusion of more female vaccinators in the health workforce. Due to sociocultural and gender norms in underserved communities of LMICs such as Pakistan, only female frontline health workers have unrestricted access to households, are able to interact with mothers and provide health education, and deliver vaccines to children. More female vaccinators could, therefore, promote building trust concerning vaccines and encourage immunization uptake among vulnerable communities. To enhance the female position in the immunization decision-making process for their children, we must focus on overall education for females, specifically in health literacy. A previous study revealed that females who were health literate, regardless of their educational level, were more likely to vaccinate their children, in both rural and urban settings [37]. Additionally, female groups in local settings and communities can be initiated or leveraged as a platform for counseling focused on health literacy. These groups could be complemented with programs to involve fathers, including facilitating regular sessions with females and males to foster collaborative parenting and decision-making. A gender-centric approach to the overall health system should be strengthened by measures such as separate waiting areas for females in immunization clinics and the introduction of female-only transport to immunization centers, which could increase immunization rates among females. 

Our study had a few limitations. The adjusted M:F ratios reported in the analyses represented a best-guess given the lack of reliable sex ratios in birth data at district and UC levels. Although 1.055 represented an aggregate number for the country, this masked the heterogeneity and inequities in M:F ratios across districts and union councils. Additionally, studies have shown that sex ratios at birth varied by levels of maternal education [38,39,40], ethnicity, the birth order of the child, as well as the economic and cultural heterogeneities [41]. Therefore, adjustments using aggregate M:F ratios at birth could mask the true extent of prevailing inequities at the sub-national level. Moreover, the M:F ratios calculated for maternal literacy, geographic location, and mode of vaccination delivery were not adjusted for the underlying proportion of males and females in the population due to the unavailability of baseline population proportions for these categories. Nonetheless, we speculated that even if these were to be adjusted, the high M:F ratios (up to 5.00 at the UC level) still reflected substantial inequities between females and males. To validate our estimates of M:F ratios further, we correlated them with the gender-wise proportions in the Multiple Indicators Cluster Survey (MICs). However, since the sample size in the MICs was small when compared against our analysis categories, no meaningful, statistically significant correlations were found between the gender proportions in our analysis and MICs. Additionally, only 58% of the remote-rural UCs in the province, as per our source, were matched with the UC database in SEIR due to different names and a variation in UC categorization used by the health and education departments. Lastly, we acknowledge that a long-term horizon of four years to observe inequity trends did not account for various factors that typically change over time (district-level government staff including supervisors and vaccinators, external shocks such as COVID-19, and unprecedented flooding), and these may have confounded the impact of the gender-based inequities over the last four years. However, by also focusing on regions that have persistently demonstrated worse immunization outcomes for females, we showed the deep-seated inequities that continue to persist despite external changes over time.

## 5. Conclusions

Our study demonstrated evidence of the gender-based inequities in Sindh Province, Pakistan, over the last four years, with a higher number of males than females being enrolled and immunized. Once enrolled, the coverage rates of females and males were similar, although females tended to be delayed in receiving their vaccinations, as compared to males. We also observed geographical pockets where females continued to fall behind males, year-on-year, reflecting the persistent nature of the inequalities. Our findings have important implications for the inequities among zero-dose children who are more likely to be females. We also demonstrated that certain factors such as maternal literacy, place of residence, and supply-side factors (mode of vaccination delivery, gender of vaccinators), were both a cause and consequence of gender-based inequities. Socio-cultural factors are inextricably linked to characteristics that lead to poor immunization outcomes for females. A deeper qualitative investigation at the sub-national level is needed to uncover the complex dynamics that impact equities in coverage, so that tailored and targeted strategies can be implemented to ensure females and males have the same opportunities to access and benefit from life-saving immunizations.

## Figures and Tables

**Table 1:** District-wise adjusted male-to-female ratios of 0–23-month-old children in 2019–2022 birth cohorts enrolled in SEIR and vaccinated, by district (*n* = 6,235,305) (1 January 2019–31 December 2022).

					At Vaccination								
Enrollment/ Vaccination District	# of UCs	Enrollment	Median (IQR)	UC Range	Penta-1	Median (IQR)	UC Range	Penta-3	Median (IQR)	UC Range	Measles-1	Median (IQR)	UC Range
Badin	46	1.05	1.02 (0.98–1.11)	0.89–1.55	1.05	1.03 (0.98–1.11)	0.89–1.55	1.06	1.02 (0.99–1.12)	0.90–1.57	1.06	1.03 (0.97–1.11)	0.90–1.64
Dadu	52	1.04	1.02 (0.96–1.09)	0.80–1.20	1.04	1.02 (0.98–1.09)	0.82–1.19	1.04	1.03 (0.99–1.09)	0.83–1.21	1.04	1.04 (0.99–1.09)	0.82–1.22
Ghotki	40	1.1	1.11 (1.04–1.16)	0.90–1.42	1.1	1.10 (1.04–1.15)	0.90–1.40	1.1	1.09 (1.04–1.16)	0.93–1.39	1.09	1.10 (1.04–1.14)	0.93–1.33
Hyderabad	54	1	1.02 (0.96–1.05)	0.69–1.26	1	1.01 (0.95–1.05)	0.73–1.28	0.99	1.01 (0.95–1.04)	0.69–1.35	1	1.01 (0.94–1.05)	0.68–1.31
Jacobabad	40	1.07	1.07 (1.00–1.13)	0.89–1.41	1.07	1.06 (1.01–1.13)	0.86–1.40	1.08	1.07 (1.01–1.17)	0.89–1.35	1.08	1.07 (1.01–1.17)	0.91–1.28
Jamshoro	28	1.04	1.02 (0.97–1.13)	0.85–2.26	1.04	1.02 (0.97–1.10)	0.86–2.03	1.04	1.01 (0.96–1.10)	0.83–1.78	1.04	1.03 (0.97–1.10)	0.85–1.77
Kambar	40	1	1.02 (0.95–1.05)	0.71–1.20	1	1.02 (0.96–1.06)	0.79–1.22	1	1.01 (0.98–1.05)	0.77–1.22	0.99	1.00 (0.97–1.04)	0.75–1.19
Karachi Central	51	0.99	0.98 (0.93–1.03)	0.63–1.33	0.99	0.98 (0.94–1.02)	0.63–1.28	0.99	0.98 (0.95–1.03)	0.59–1.19	0.99	0.99 (0.95–1.03)	0.60–1.19
Karachi East	28	0.98	0.99 (0.94–1.02)	0.85–1.08	0.99	1.00 (0.96–1.03)	0.85–1.10	0.98	0.98 (0.96–1.01)	0.84–1.09	0.98	0.99 (0.94–1.03)	0.90–1.12
Karachi South	26	0.99	0.99 (0.97–1.03)	0.91–1.11	1	1.01 (0.98–1.04)	0.91–1.20	1	1.01 (0.98–1.04)	0.89–1.09	1.01	1.02 (0.97–1.05)	0.90–1.15
Karachi West	22	0.98	0.97 (0.95–1.03)	0.88–1.06	0.98	0.97 (0.96–1.02)	0.89–1.07	0.97	0.98 (0.95–1.03)	0.87–1.06	0.97	0.96 (0.95–1.03)	0.86–1.04
Kashmore	37	1.15	1.11 (1.04–1.25)	0.92–1.56	1.14	1.12 (1.06–1.22)	0.95–1.46	1.13	1.12 (1.04–1.21)	0.91–1.35	1.12	1.11 (1.05–1.22)	0.90–1.36
Kemari	21	0.98	0.98 (0.96–1.01)	0.83–1.17	0.99	0.98 (0.96–1.02)	0.85–1.13	0.98	0.99 (0.96–1.03)	0.86–1.10	0.99	1.00 (0.96–1.03)	0.85–1.09
Khairpur	76	1.06	1.03 (1.00–1.11)	0.83–1.41	1.06	1.04 (1.00–1.12)	0.86–1.36	1.07	1.05 (1.00–1.12)	0.88–1.41	1.07	1.04 (0.99–1.12)	0.88–1.38
Korangi	30	1.01	1.02 (0.98–1.04)	0.86–1.29	1.01	1.02 (0.99–1.04)	0.89–1.24	1	1.01 (0.99–1.02)	0.90–1.18	1	1.00 (0.98–1.03)	0.89–1.17
Larkana	46	1.04	1.03 (0.97–1.07)	0.89–1.58	1.04	1.03 (0.98–1.08)	0.90–1.49	1.05	1.04 (1.01–1.09)	0.90–1.46	1.05	1.03 (0.98–1.11)	0.91–1.45
Malir	19	0.99	1.00 (0.97–1.02)	0.94–1.03	1	1.00 (0.98–1.02)	0.94–1.05	1	1.00 (0.97–1.02)	0.95–1.05	1	1.01 (0.99–1.02)	0.85–1.04
Matiari	18	1.1	1.06 (1.01–1.10)	0.95–1.53	1.1	1.05 (1.02–1.11)	0.94–1.51	1.1	1.05 (1.03–1.11)	0.94–1.58	1.08	1.04 (1.02–1.11)	0.93–1.51
Mirpurkhas	41	1.03	1.02 (0.98–1.06)	0.93–1.27	1.03	1.03 (0.97–1.07)	0.93–1.25	1.03	1.02 (0.99–1.07)	0.90–1.31	1.02	1.01 (0.98–1.06)	0.91–1.29
Naushero Feroz	51	1.04	1.05 (0.97–1.11)	0.89–1.22	1.05	1.05 (0.97–1.10)	0.92–1.21	1.05	1.05 (0.98–1.09)	0.93–1.23	1.04	1.01 (0.99–1.08)	0.92–1.23
Sanghar	55	1.09	1.08 (1.00–1.15)	0.82–1.37	1.09	1.08 (1.00–1.14)	0.87–1.38	1.08	1.06 (1.02–1.15)	0.95–1.32	1.08	1.08 (1.01–1.15)	0.94–1.28
Shaheed Benazirabad	51	1.05	1.06 (1.01–1.10)	0.92–1.24	1.04	1.06 (1.01–1.10)	0.93–1.23	1.04	1.04 (1.01–1.09)	0.93–1.21	1.04	1.04 (1.00–1.11)	0.92–1.22
Shikarpur	49	1.02	1.02 (0.97–1.07)	0.88–1.22	1.02	1.01 (0.98–1.07)	0.89–1.21	1.01	1.01 (0.97–1.06)	0.86–1.20	1.01	1.00 (0.97–1.05)	0.89–1.21
Sujawal	25	1.05	1.06 (0.97–1.09)	0.89–1.33	1.05	1.06 (0.98–1.09)	0.89–1.30	1.04	1.04 (0.98–1.09)	0.89–1.29	1.04	1.03 (0.98–1.09)	0.89–1.26
Sukkur	46	1.08	1.05 (1.02–1.11)	0.71–1.82	1.07	1.05 (1.03–1.13)	0.76–1.63	1.07	1.07 (1.01–1.11)	0.80–1.47	1.07	1.07 (1.01–1.11)	0.84–1.47
Tando Allahyar	20	1.06	1.05 (1.00–1.10)	0.93–1.23	1.06	1.03 (1.01–1.09)	0.96–1.21	1.05	1.05 (1.01–1.09)	0.95–1.19	1.05	1.05 (1.02–1.09)	0.95–1.14
Tando Muhammad Khan	17	1.02	1.03 (0.97–1.10)	0.72–1.40	1.02	1.02 (0.98–1.11)	0.73–1.41	1.03	1.02 (1.00–1.09)	0.71–1.40	1.03	1.04 (0.99–1.10)	0.69–1.36
Tharparkar	44	1.11	1.12 (1.02–1.18)	0.85–1.69	1.11	1.11 (1.03–1.17)	0.85–1.68	1.11	1.13 (1.02–1.17)	0.85–1.65	1.11	1.12 (1.03–1.19)	0.86–1.55
Thatta	30	1.09	1.07 (1.02–1.12)	0.75–3.00	1.09	1.06 (1.02–1.11)	0.76–2.85	1.09	1.07 (1.02–1.12)	0.75–2.82	1.08	1.06 (1.02–1.11)	0.71–2.73
Umerkot	27	1.05	1.06 (1.01–1.11)	0.90–1.21	1.05	1.05 (1.00–1.10)	0.91–1.18	1.05	1.05 (0.98–1.09)	0.92–1.20	1.03	1.03 (0.98–1.05)	0.92–1.14
Total	1130	1.04	1.03 (0.98–1.10)	0.63–3.00	1.04	1.04 (0.98–1.10)	0.63–2.85	1.04	1.03 (0.99–1.09)	0.59–2.82	1.04	1.03 (0.98–1.09)	0.60–2.73

#: number.

**Table 2:** Male-to-Female ratios of 0–23-month-old children in 2019–2022 birth cohorts enrolled in SEIR in Sindh Province, Pakistan, by maternal literacy levels, geographic profile, vaccination modality, and vaccinator sex ratio at enrollment for Pentavalent-1, Pentavalent-3 and Measles-1 vaccines (*n* = 6,235,305) (1 January 2019–31 December 2022).

	Enrollment			Pentavalent-1			Pentavalent-3			Measles-1		
	M:F Ratio	Median (IQR)	UC Range	M:F Ratio	Median (IQR)	UC Range	M:F Ratio	Median (IQR)	UC Range	M:F Ratio	Median (IQR)	UC Range
Mother’s education (years)												
0	1.09	1.06 (0.93–1.21)	0.00–5.00	1.09	1.04 (0.82–1.33)	0.00–11.00	1.09	1.05 (0.86–1.38)	0.00–9.00	1.08	1.04 (0.85–1.35)	0.00–7.00
1–5	1.11	1.04 (0.84–1.24)	0.00–8.00	1.11	1.09 (0.92–1.30)	0.00–7.00	1.11	1.10 (0.91–1.33)	0.00–9.00	1.11	1.10 (0.93–1.33)	0.00–5.00
6–8	1.12	1.00 (0.67–1.35)	0.00–8.00	1.12	1.00 (0.50–1.33)	0.00–7.00	1.12	1.00 (0.50–1.38)	0.00–7.00	1.12	1.00 (0.50–1.33)	0.00–11.00
9–10	1.09	1.00 (0.54–1.27)	0.00–7.00	1.09	1.00 (0.50–1.37)	0.00–9.00	1.10	1.00 (0.50–1.42)	0.00–9.00	1.10	1.00 (0.50–1.47)	0.00–10.00
≥11	1.07	1.00 (0.50–1.33)	0.00–10.00	1.07	1.00 (0.33–1.33)	0.00–11.00	1.07	1.00 (0.50–1.25)	0.00–6.00	1.07	1.00 (0.25–1.33)	0.00–7.00
Geographic location												
Rural	1.12	1.11 (1.06–1.17)	0.00–2.38	1.12	1.11 (1.06–1.17)	0.50–2.14	1.12	1.11 (1.06–1.18)	0.50–1.80	1.12	1.10 (1.05–1.17)	0.00–2.17
Urban	1.07	1.07 (1.02–1.11)	0.00–2.37	1.07	1.06 (1.02–1.11)	0.50–2.50	1.07	1.06 (1.02–1.11)	0.00–2.61	1.07	1.07 (1.02–1.12)	0.44–5.00
Remote-rural	1.15	1.14 (1.08–1.21)	0.71–2.07	1.15	1.14 (1.07–1.21)	0.69–1.77	1.15	1.13 (1.07–1.22)	0.94–1.74	1.15	1.13 (1.08–1.22)	0.80–1.75
Rural	1.12	1.10 (1.05–1.17)	0.00–2.38	1.12	1.10 (1.06–1.16)	0.50–2.14	1.12	1.11 (1.06–1.17)	0.50–1.80	1.11	1.10 (1.05–1.17)	0.00–2.17
Slums	1.08	1.07 (1.03–1.11)	0.00–2.37	1.08	1.07 (1.03–1.11)	0.50–2.50	1.08	1.07 (1.03–1.12)	0.00–2.61	1.08	1.07 (1.03–1.12)	0.44–5.00
Non-slums	1.05	1.05 (1.02–1.08)	0.83–1.76	1.05	1.05 (1.01–1.09)	0.81–1.70	1.05	1.05 (1.02–1.09)	0.82–1.56	1.05	1.06 (1.01–1.09)	0.90–2.38
Vaccination modality												
Fixed	1.09	1.03–1.17	0.00–8.00	1.09	1.09 (1.03–1.18)	0.00–4.18	1.09	1.09 (1.02–1.19)	0.00–3.50	1.09	1.09 (1.01–1.19)	0.00–6.00
Outreach	1.09	1.01–1.17	0.54–3.25	1.09	1.08 (1.01–1.16)	0.00–3.51	1.10	1.09 (1.02–1.17)	0.00–3.51	1.10	1.08 (1.02–1.16)	0.00–3.67
EOA	1.09	1.00–1.17	0.00–3.08	1.08	1.07 (0.99–1.17)	0.00–5.00	1.09	1.07 (1.00–1.17)	0.00–6.00	1.09	1.07 (1.00–1.16)	0.00–5.00
Vaccinators’ sex												
No female vaccinator	1.11	1.00 (1.00–1.20)	0.80–3.00	1.11	1.20 (1.00–1.20)	0.80–3.00	1.11	1.00 (1.00–1.20)	0.80–2.80	1.11	1.00 (1.00–1.20)	0.80–2.80
M:F ratio ≥ 2	1.08	1.00 (1.00–1.20)	0.80–1.60	1.08	1.00 (1.00–1.20)	1.00–1.60	1.07	1.00 (1.00–1.20)	0.80–1.40	1.08	1.00 (1.00–1.20)	1.00–1.40
1 < M:F ratio < 2	1.07	1.00 (1.00–1.20)	0.80–1.20	1.07	1.00 (1.00–1.20)	1.00–1.20	1.06	1.00 (1.00–1.00)	1.00–1.20	1.06	1.00 (1.00–1.00)	1.00–1.20
M:F ratio = 1	1.06	1.00 (1.00–1.00)	0.60–1.80	1.05	1.00 (1.00–1.00)	0.60–1.60	1.05	1.00 (1.00–1.00)	0.60–1.60	1.05	1.00 (1.00–1.00)	0.60–1.60
M:F ratio < 1	1.03	1.00 (1.00–1.00)	1.00–1.20	1.04	1.00 (1.00–1.00)	1.00–1.20	1.04	1.00 (1.00–1.00)	1.00–1.20	1.04	1.00 (1.00–1.00)	1.00–1.20
No vaccinator	1.16	1.20 (1.20–1.20)	1.20–1.20	1.25	1.20 (1.20–1.20)	1.20–1.20	1.01	1.00 (1.00–1.00)	1.00–1.00	1.21	1.20 (1.20–1.20)	1.20–1.20

## Data Availability

Data may be obtained from a third party and are not publicly available. The data used for this analysis from Sindh Electronic Immunization Registry (SEIR; also known as Zindagi Mehfooz program) can be requested from the Government of Sindh’s Expanded Programme on Immunization (EPI).

## Supplementary Materials

The following supporting information can be downloaded at: https://www.mdpi.com/article/10.3390/vaccines11030685/s1, Figure S1: Location of Urban, Rural, and Remote-Rural UCs in Sindh Province, Pakistan (*n* = 1130); Figure S2: Annual male-to-female ratios among children (>6 weeks) who received Penta-1, Penta-3, and Measles-1 vaccinations in 2019–2022 birth cohorts enrolled in SEIR (1 January 2019–31 December 2022); Figure S3: Annual gender inequality ratios (GIR) among children (>6 weeks) who received Penta-1, Penta-3, and Measles-1 vaccinations in 2019–2022 birth cohorts enrolled in SEIR (1 January 2019–31 December 2022); Figure S4: Geographical distribution of UCs showing annual male-to-female ratios of >1.10 among children (>6 weeks) who received Penta-1, Penta-3, and Measles-1 vaccinations in 2019–2022 birth cohorts enrolled in SEIR (1 January 2019–31 December 2022); Figure S5: Location of UCs with differing sex ratios of vaccinators in Sindh Province, Pakistan (*n* = 3354); Table S1: Gender inequality ratios of 0–23-month-old children in 2019–2022 birth cohorts in SEIR at enrollment and vaccination coverage in Sindh Province, Pakistan, by district (*n* = 6,235,305) (1 January 2019–31 December 2022); Table S2: Gender inequality ratios of 0–23-month-old children in 2019–2022 birth cohorts in SEIR at enrollment and by antigens by maternal literacy levels, geographic location, vaccinators sex ratio, and modality of immunization delivery (*n* = 6,235,305) (1 January 2019–31 December 2022); Table S3: Number of UCs showing persistent gender inequities in vaccination (M:F > 1.05) from 2019–2022 (*n* = 1129);Table S4: Categorization of remote-rural, rural, and urban UCs by sex ratio of vaccinators (*n* = 1130).
