# Supplementary material for: Immunization Gender Inequity in Pakistan: An Analysis of 6.2 Million Children Born from 2019 to 2022 and Enrolled in the Sindh Electronic Immunization Registry
Source: Vaccines (Basel). 2023 Mar 17;11(3):685. doi: 10.3390/vaccines11030685 (PMC10059986; doi:10.3390/vaccines11030685)
Supplement: Supplementary file 1 [file vaccines-11-00685-s001.zip › vaccines-2254688-supplementary.pdf]

Supplementary

Figure S1: Location of Urban, Rural, and Remote-Rural UCs in Sindh Province, Pakistan (*n* = 1130)

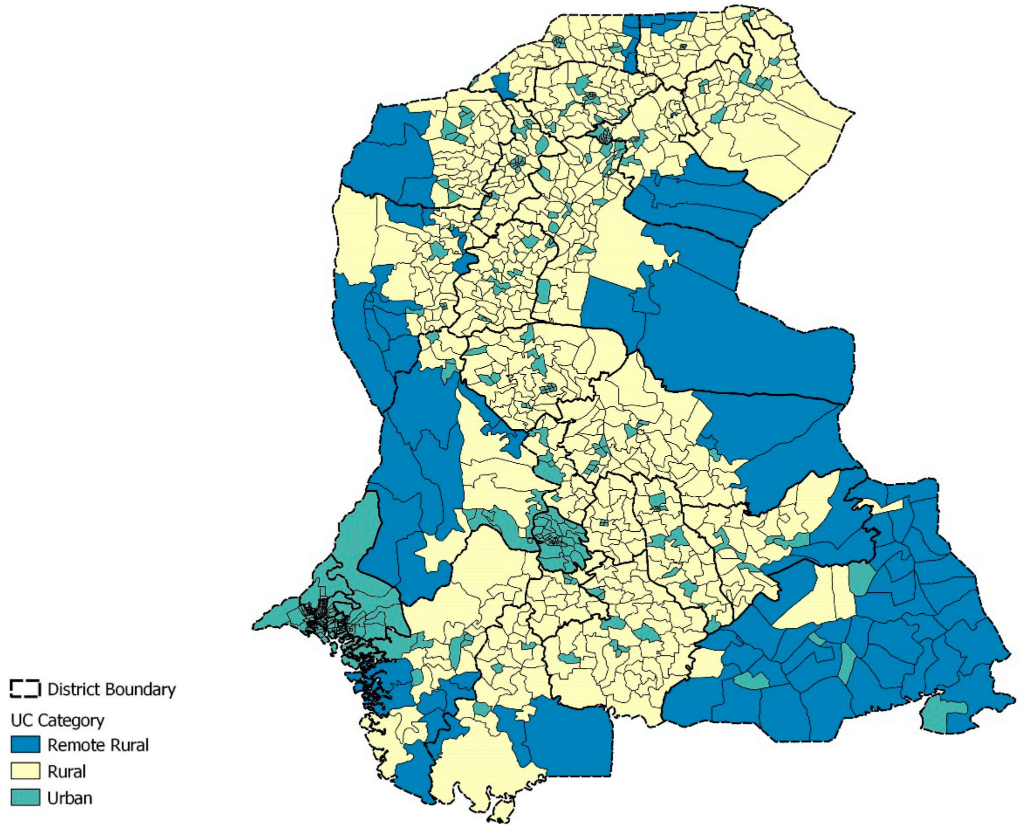

Figure S2: Annual male-to-female ratios among children (>6 weeks) who received Penta-1, Penta-3, and Measles-1 vaccinations in 2019–2022 birth cohorts enrolled in SEIR (1 January 2019–31 December 2022)

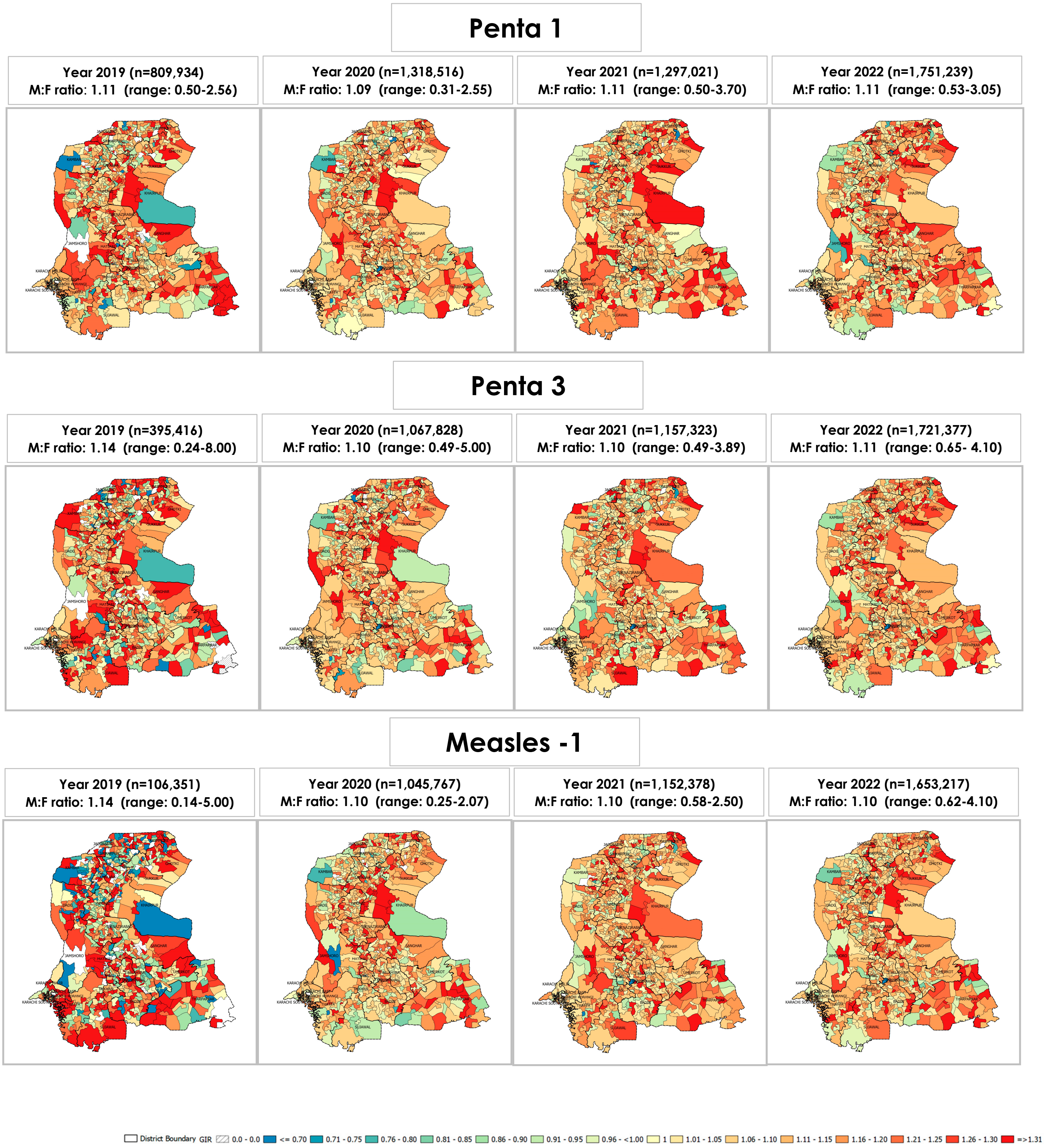

Figure S3: Annual gender inequality ratios (GIR) among children (>6 weeks) who received Penta-1, Penta-3, and Measles-1 vaccinations in 2019–2022 birth cohorts enrolled in SEIR (1 January 2019–31 December 2022)

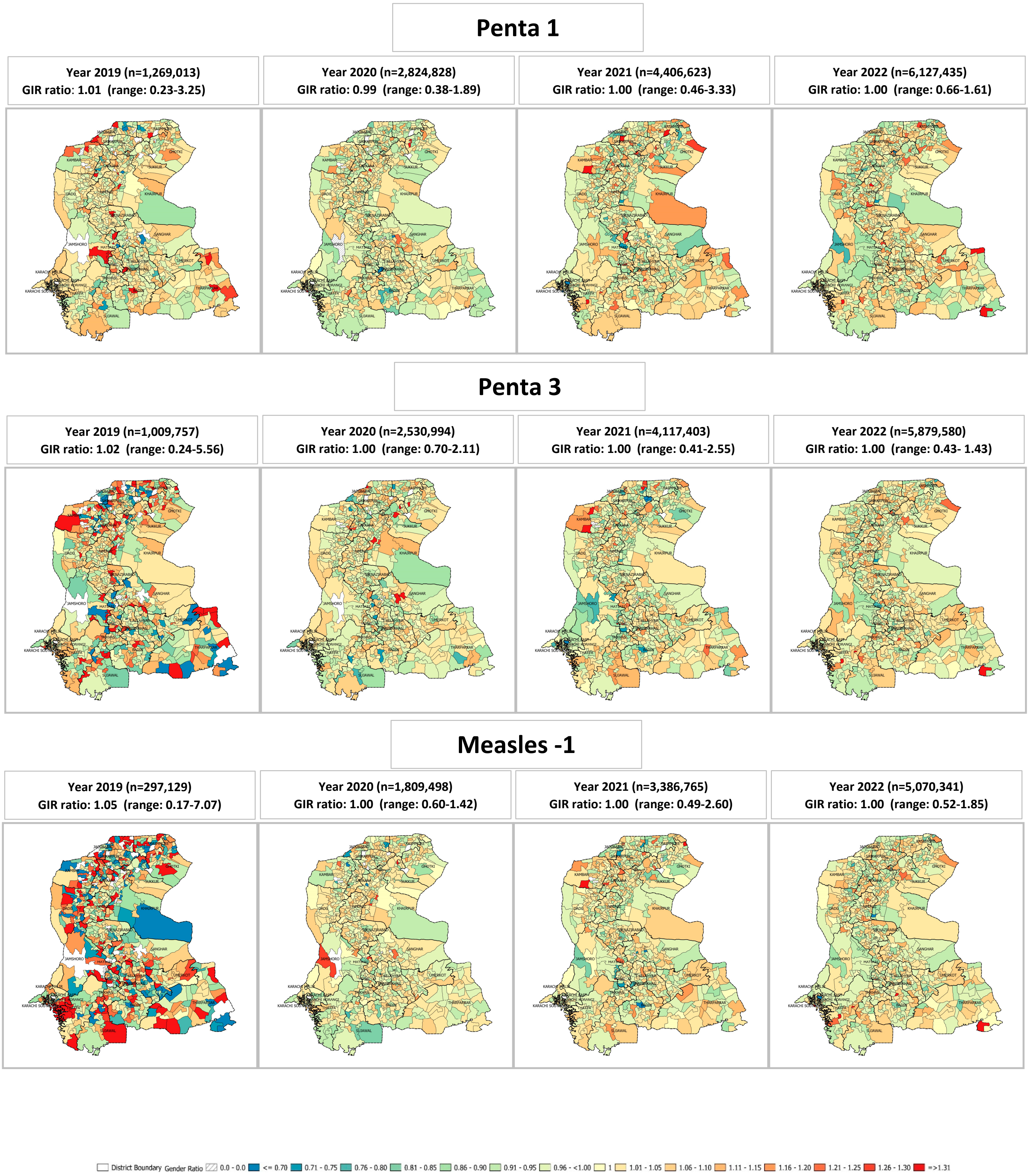

Figure S4: Geographical distribution of UCs showing annual male-to-female ratios of >1.10 among children (>6 weeks) who received Penta-1, Penta-3, and Measles-1 vaccinations in 2019–2022 birth cohorts enrolled in SEIR (1 January 2019–31 December 2022)

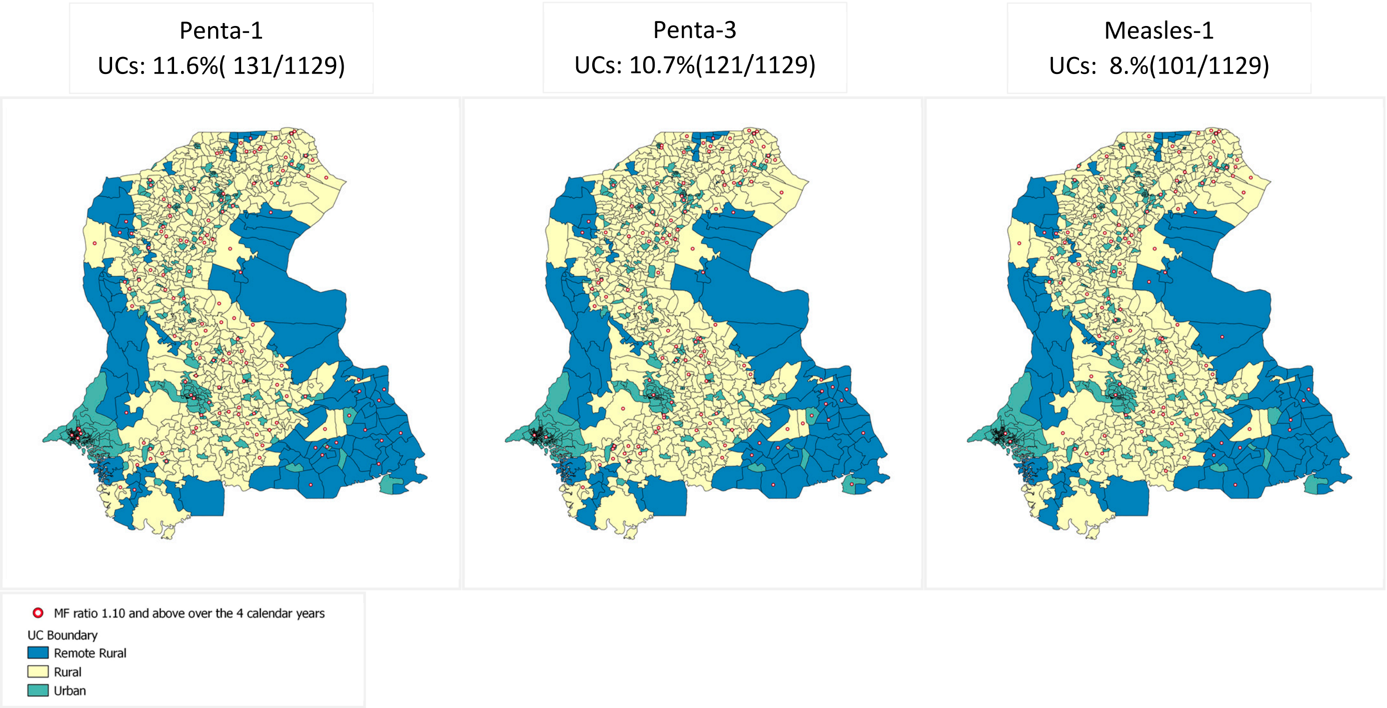

Figure S5: Location of UCs with differing sex ratios of vaccinators in Sindh Province, Pakistan (n = 3354)

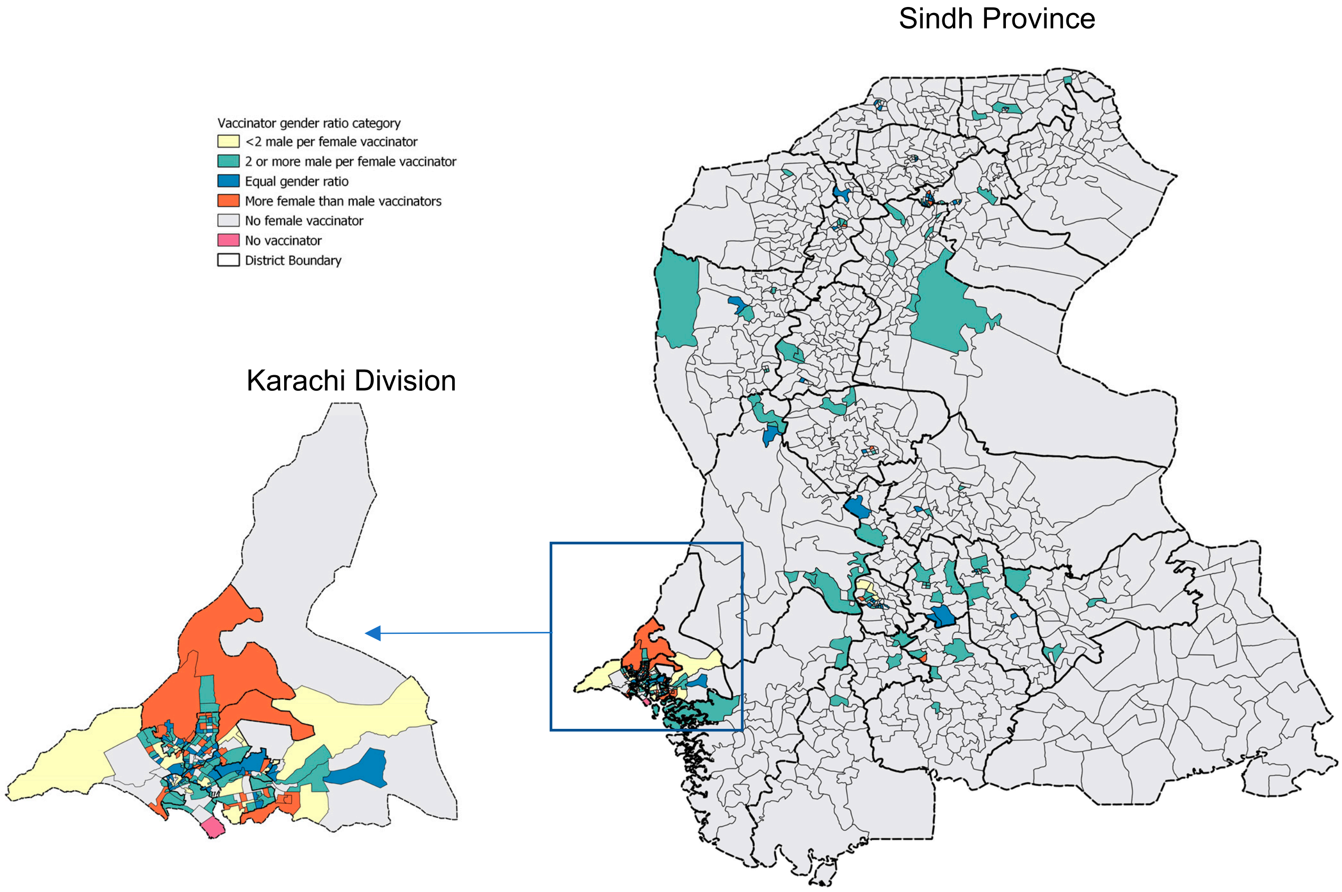

Table S1: Gender inequality ratios of 0–23-month-old children in 2019–2022 birth cohorts in SEIR at enrollment and vaccination coverage in Sindh Province, Pakistan, by district (*n* = 6,235,305) (1 January 2019–31 December 2022);

Residential district	# of UCs	Pentavalent-1 vaccination	Median (IQR)	UC range	Pentavalent-3 vaccination	Median (IQR)	UC range	Measles-1 vaccination	Median (IQR)	UC range
Badin	46	1.00	1.00 (1.00-1.00)	0.99-1.01	1.00	1.01 (1.00-1.01)	0.96-1.05	1.00	1.00 (0.99-1.01)	0.97-1.04
Dadu	52	1.00	1.00 (1.00-1.00)	0.99-1.02	1.00	1.00 (1.00-1.01)	0.97-1.04	1.00	1.00 (1.00-1.01)	0.98-1.03
Ghotki	40	1.00	1.00 (1.00-1.00)	0.99-1.02	1.00	1.00 (0.99-1.01)	0.97-1.03	1.00	1.00 (0.99-1.01)	0.94-1.03
Hyderabad	54	1.00	1.00 (0.99-1.01)	0.98-1.02	0.99	0.99 (0.99-1.00)	0.96-1.04	1.00	1.00 (0.99-1.01)	0.95-1.04
Jacobabad	40	1.00	1.00 (0.99-1.00)	0.97-1.03	1.01	1.01 (0.99-1.02)	0.92-1.07	1.01	1.00 (0.99-1.02)	0.95-1.07
Jamshoro	28	1.00	1.00 (0.99-1.01)	0.97-1.02	1.00	1.00 (0.99-1.01)	0.91-1.02	0.99	1.00 (0.98-1.01)	0.95-1.03
Kambar	40	1.00	1.00 (0.99-1.01)	0.98-1.02	1.00	1.00 (0.98-1.02)	0.96-1.05	1.00	1.00 (0.98-1.02)	0.93-1.05
Karachi Central	51	1.00	1.00 (1.00-1.00)	0.96-1.02	1.00	1.00 (0.99-1.01)	0.92-1.04	1.00	1.00 (0.99-1.01)	0.92-1.06
Karachi East	28	1.00	1.00 (0.99-1.01)	0.99-1.02	1.00	1.00 (0.99-1.01)	0.96-1.05	1.00	1.00 (0.99-1.02)	0.97-1.06
Karachi South	26	1.00	1.00 (1.00-1.01)	0.98-1.03	1.00	1.00 (0.99-1.02)	0.96-1.05	1.01	1.00 (0.99-1.02)	0.96-1.06
Karachi West	22	1.00	1.00 (1.00-1.01)	0.99-1.02	1.00	1.00 (0.98-1.01)	0.97-1.03	1.00	1.00 (0.98-1.01)	0.97-1.03
Kashmore	37	1.00	1.00 (0.99-1.00)	0.97-1.05	0.99	0.99 (0.97-1.01)	0.90-1.06	0.99	1.00 (0.98-1.01)	0.95-1.02
Kemari	21	1.00	1.00 (1.00-1.01)	0.99-1.04	1.00	1.00 (0.99-1.01)	0.94-1.03	1.00	1.00 (0.99-1.02)	0.97-1.05
Khairpur	76	1.00	1.00 (1.00-1.00)	0.98-1.03	1.00	1.00 (0.99-1.01)	0.95-1.03	1.00	1.00 (0.99-1.01)	0.97-1.04
Korangi	30	1.00	1.00 (1.00-1.00)	0.95-1.01	1.00	1.00 (0.99-1.00)	0.57-1.01	1.00	0.99 (0.99-1.00)	0.96-2.11
Larkana	46	1.00	1.00 (1.00-1.01)	0.99-1.02	1.01	1.01 (1.00-1.02)	0.97-1.05	1.01	1.01 (1.00-1.03)	0.96-1.04
Malir	19	1.00	1.00 (1.00-1.01)	0.99-1.02	1.01	1.00 (1.00-1.02)	0.99-1.04	1.01	1.00 (0.99-1.03)	0.98-1.15
Matiari	18	1.00	1.00 (1.00-1.01)	0.99-1.02	1.00	1.01 (0.99-1.02)	0.96-1.08	1.00	1.01 (0.99-1.01)	0.96-1.09
Mirpurkhas	41	1.00	1.00 (1.00-1.01)	0.98-1.03	1.00	1.00 (0.99-1.01)	0.95-1.07	1.00	1.00 (0.99-1.01)	0.97-1.03
Naushero Feroz	51	1.00	1.00 (1.00-1.01)	0.99-1.03	1.00	1.00 (0.99-1.01)	0.96-1.05	1.00	1.00 (0.99-1.01)	0.97-1.06
Sanghar	55	1.00	1.00 (1.00-1.00)	0.98-1.02	1.00	1.00 (0.99-1.01)	0.94-1.02	1.00	1.00 (0.99-1.01)	0.97-1.02
Shaheed Benazirabad	51	1.00	1.00 (1.00-1.00)	0.98-1.02	1.00	1.00 (0.99-1.01)	0.98-1.03	1.00	1.00 (0.99-1.01)	0.98-1.03
Shikarpur	49	1.00	1.00 (0.99-1.00)	0.98-1.02	0.99	0.99 (0.98-1.00)	0.95-1.04	1.00	0.99 (0.98-1.00)	0.96-1.04
Sujawal	25	1.00	1.00 (1.00-1.01)	0.99-1.01	0.99	0.99 (0.98-1.02)	0.96-1.07	0.99	1.00 (0.99-1.01)	0.96-1.03
Sukkur	46	1.00	1.00 (0.99-1.01)	0.97-1.03	0.99	1.00 (0.98-1.01)	0.94-1.06	1.00	1.00 (0.99-1.02)	0.93-1.05
Tando Allahyar	20	1.00	1.00 (1.00-1.01)	0.98-1.01	1.00	1.00 (0.98-1.00)	0.96-1.03	1.00	1.00 (0.99-1.01)	0.95-1.03
Tando Muhammad Khan	17	1.00	1.00 (1.00-1.01)	0.99-1.01	1.01	1.00 (1.00-1.01)	0.98-1.05	1.00	1.00 (0.99-1.00)	0.98-1.02
Tharparkar	44	1.00	1.00 (1.00-1.00)	0.99-1.02	1.00	1.00 (0.99-1.01)	0.93-1.02	1.00	1.00 (0.99-1.00)	0.96-1.02
Thatta	30	1.00	1.00 (0.99-1.01)	0.99-1.04	1.00	1.00 (0.99-1.01)	0.94-1.08	1.00	1.00 (0.99-1.01)	0.97-1.02
Umerkot	27	1.00	1.00 (0.99-1.00)	0.98-1.01	1.00	1.00 (0.98-1.01)	0.97-1.04	1.00	0.99 (0.98-1.01)	0.97-1.05
**Total**	**1,130**	**1.00**	**1.00 (1.00-1.01)**	**0.95-1.05**	**1.00**	**1.00 (0.99-1.01)**	**0.57-1.08**	**1.00**	**1.00 (0.99-1.01)**	**0.92-2.11**

Table S2: Gender inequality ratios of 0–23-month-old children in 2019–2022 birth cohorts in SEIR at enrollment and by antigens by maternal literacy levels, geographic location , vaccinators sex ratio , and modality of immunization delivery (*n* = 6,235,305) (1 January 2019–31 December 2022)

	Pentavalent-1			Pentavalent-3			Measles-1		
	**GIR**	**Median (IQR)**	**UC range**	**GIR**	**Median (IQR)**	**UC range**	**GIR**	**Median (IQR)**	**UC range**
**Maternal literacy level**									
Illiterate	1.00	1.00 (1.00-1.00)	0.00-2.00	1.00	1.00 (0.98-1.02)	0.00-3.12	GIR	1.00 (0.98-1.01)	0.00-3.20
1-5	1.00	1.00 (1.00-1.00)	0.00-2.00	1.00	1.00 (0.97-1.03)	0.00-2.50	1.00	1.00 (0.97-1.03)	0.00-3.00
6-8	1.00	1.00 (1.00-1.01)	0.00-3.57	1.00	1.00 (0.93-1.01)	0.00-3.18	1.00	1.00 (0.95-1.05)	0.00-3.75
9-10	1.00	1.00 (1.00-1.00)	0.00-2.00	1.01	1.00 (0.96-1.06)	0.00-2.63	1.01	1.00 (0.94-1.06)	0.00-4.00
>=11	1.00	1.00 (1.00-1.00)	0.00-1.50	1.00	1.00 (0.93-1.04)	0.00-3.00	1.00	1.00 (0.93-1.04)	0.00-3.20
**Geographic profile**									
Rural	1.00	1.00 (1.00-1.00)	0.97-1.05	1.00	1.00 (0.99-1.01)	0.90-1.08	1.00	1.00 (0.99-1.01)	0.93-1.09
Urban	1.00	1.00 (1.00-1.01)	0.95-1.04	1.00	1.00 (0.99-1.01)	0.57-1.08	1.00	1.00 (0.99-1.01)	0.92-2.11
Remote rural	1.00	1.00 (1.00-1.00)	0.97-1.03	1.00	1.00 (0.99-1.02)	0.93-1.06	1.00	1.00 (0.99-1.00)	0.95-1.04
Rural	1.00	1.00 (1.00-1.01)	0.97-1.05	1.00	1.00 (0.99-1.01)	0.90-1.08	1.00	1.00 (0.99-1.01)	0.93-1.09
Slum	1.00	1.00 (1.00-1.01)	0.95-1.04	1.00	1.00 (0.99-1.01)	0.57-1.08	1.00	1.00 (0.99-1.01)	0.92-2.11
Non-slum	1.00	1.00 (1.00-1.01)	0.98-1.04	1.00	1.00 (0.99-1.01)	0.94-1.04	1.00	1.00 (0.99-1.01)	0.96-1.04
**Vaccination modality**									
Fixed	1.00	1.01 (0.97-1.05)	0.36-4.14	1.00	1.01 (0.96-1.06)	0.46-2.40	1.00	1.01 (0.95-1.07)	0.13-4.77
Outreach	1.00	1.00 (0.96-1.03)	0.55-1.83	1.01	1.00 (0.97-1.03)	0.76-1.59	1.00	1.00 (0.96-1.03)	0.53-2.03
EOA	0.99	0.99 (0.93-1.04)	0.24-4.37	0.99	0.99 (0.93-1.05)	0.21-6.09	1.00	0.99 (0.93-1.04)	0.14-5.29
**Vaccinators' gender ratio categories**									
No female vaccinator	1.00	1.00 (1.00-1.00)	0.97-1.05	1.00	1.00 (0.99-1.01)	0.90-1.08	1.00	1.00 (0.99-1.01)	0.93-1.15
M:F ratio >=2	1.00	1.00 (1.00-1.01)	0.98-1.03	1.00	1.00 (0.99-1.01)	0.96-1.07	1.00	1.00 (0.99-1.01)	0.96-1.06
1<M:F ratio<2	1.00	1.00 (1.00-1.01)	0.98-1.02	1.00	0.99 (0.99-1.00)	0.97-1.03	1.00	1.00 (0.99-1.00)	0.98-1.05
M:F ratio<1	1.00	1.00 (1.00-1.01)	0.99-1.02	1.00	1.00 (0.99-1.01)	0.98-1.05	1.00	1.00 (0.99-1.01)	0.97-1.06
M:F ratio=1	1.00	1.00 (0.99-1.00)	0.96-1.03	1.00	1.00 (0.98-1.01)	0.92-1.07	1.00	1.00 (0.99-1.01)	0.92-1.07
No vaccinator	1.03	1.03 (1.03-1.03)	1.03-1.03	0.99	0.99 (0.57-1.01)	0.57-1.01	1.00	1.06 (1.00-2.11)	1.00-2.11

Table S3: Number of UCs showing persistent gender inequities in vaccination (M:F > 1.05) from 2019–2022 (*n* = 1129)

M:F ratio	Penta 1			Penta 3			Measles -1		
	# of UCs	n	%	# of UCs	n	%	# of UCs	n	%
≥1.05	1,129	280	24.8	1,129	268	23.7	1,129	218	19.3
≥1.10	1,129	131	11.6	1,129	121	10.7	1,129	101	8.9
≥1.15	1,129	51	4.5	1,129	56	5.0	1,129	42	3.7
≥1.20	1,129	24	2.1	1,129	18	1.6	1,129	20	1.8
≥1.25	1,129	25	2.2	1,129	10	0.9	1,129	9	0.8
≥1.30	1,129	8	0.7	1,129	4	0.4	1,129	6	0.5
≥1.35	1,129	6	0.5	1,129	3	0.3	1,129	2	0.2
≥1.40	1,129	2	0.2	1,129	3	0.3	1,129	2	0.2
≥1.45	1,129	2	0.2	1,129	1	0.1	1,129	2	0.2
≥1.50	1,129	2	0.2	1,129	1	0.1	1,129	2	0.2
≥1.55	1,129	1	0.1	1,129	1	0.1	1,129	1	0.1
≥1.60	1,129	1	0.1	1,129	1	0.1	1,129	1	0.1
≥2.0	1,129	-	-	1,129	-	-	1,129	1	0.1

Table S4: Categorization of remote-rural, rural, and urban UCs by sex ratio of vaccinators (*n* = 1130)

Vaccinator' gender ratio categories	Category			
	Remote Rural	Rural	Urban	Total
No female vaccinator	87	548	217	852
M:F ratio >=2	0	26	108	134
1<M:F ratio<2	0	0	23	23
M:F ratio<1	0	0	39	39
M:F ratio=1	0	5	77	82
Total	87	579	464	1130
